# Long-range doublon transfer in a dimer chain induced by topology and ac fields

**DOI:** 10.1038/srep22562

**Published:** 2016-03-02

**Authors:** M. Bello, C. E. Creffield, G. Platero

**Affiliations:** 1 Instituto de Ciencias de Materiales, CSIC, Cantoblanco, E-28049, Madrid, Spain; 2 Departamento de Física de Materiales, Universidad Complutense de Madrid, E-28040, Madrid, Spain

## Abstract

The controlled transfer of particles from one site of a spatial lattice to another is essential for many tasks in quantum information processing and quantum communication. In this work we study how to induce long-range transfer between the two ends of a dimer chain, by coupling states that are localized just on the chain’s end-points. This has the appealing feature that the transfer occurs only between the end-points – the particle does not pass through the intermediate sites–making the transfer less susceptible to decoherence. We first show how a repulsively bound-pair of fermions, known as a doublon, can be transferred from one end of the chain to the other via topological edge states. We then show how non-topological surface states of the familiar Shockley or Tamm type can be used to produce a similar form of transfer under the action of a periodic driving potential. Finally we show that combining these effects can produce transfer by means of more exotic topological effects, in which the driving field can be used to switch the topological character of the edge states, as measured by the Zak phase. Our results demonstrate how to induce long range transfer of strongly correlated particles by tuning both topology and driving.

Recent experimental advances have provided reliable and tunable setups to test and explore the quantum mechanical world. Paradigmatic examples are ultracold atomic gases trapped in optical lattices and coherent semiconductor devices such as quantum dots. Much of the interest in the last few years has been focused on the long-range transfer of particles in these systems, bearing in mind potential applications in the fields of quantum information and quantum computing. Several mechanisms have been proposed to achieve this aim, including propagation along spin chains^1^ or a bipartite lattice^2^, coherent transport by adiabatic passage (CTAP)^3^,^4^,^5^,^6^, or the virtual occupation of intermediate states^7^,^8^,^9^. Harnessing the effects of topology has also recently become possible, in which edge states provide lossless transport that is protected against disorder. Key to the production of these topological insulators has been the use of time-dependent potentials to engineer the tunnelings in these lattice systems. This has allowed the production of quantum Hall states^10^,^11^, and more exotic topological systems such as the Haldane model^12^. It has also been shown that driving graphene with ac electric fields can be used to induce a semimetal insulator transition^13^. Inspired by these developments, in this work we study how the long-range transfer of particles can be achieved by combining these ingredients; topological effects and periodic driving.

Probably the most simple system that can exhibit topological effects is the one-dimensional dimer chain, or one-dimensional Su-Schrieffer-Heeger (SSH) model, originally introduced to describe solitonic effects in polymers^14^,^15^. Such a dimer chain supports edge states when it is in the topologically non-trivial phase. This is determined by the ratio between the two hopping rates, *J* and *J*′, a parameter we will call *λ* = *J*′/*J*^14^,^16^,^17^. Recently, many investigations have focused on this model and several results have been confirmed experimentally using ultracold atoms trapped in optical lattices^18^. Since these edge states form a non-local two-level system, a remarkable dynamics can occur for non-interacting particles moving on such a chain; they can directly pass from one end to the other without moving through the intermediate sites (see Supplementary Material). This direct transfer of particles between distant sites, which preserves the quantum coherence of the state, clearly has applications to quantum information processing, in which quantum states must be coherently shuttled between quantum gates and registers.

In this work we investigate how this long-range transfer of particles in a dimer chain can be produced and optimized in systems of strongly interacting fermions. In general, interactions are known to destroy the topological effects in the non-interacting dimer chain (Supplementary Material). However, by considering the strongly-interacting limit, in which fermions form repulsively-bound pairs called “doublons”^19^,^20^,^21^, we show that the effect can be recovered by tuning local potentials at the end-points of the lattice. We further show that driving the system with a high-frequency potential allows the manipulation of the doublon tunneling rates via the phenomenon known as coherent destruction of tunneling^22^, permitting an alternative form of long-range transfer to occur via a non-topological mechanism that we term “Shockley transfer”. Finally we show how combining lattice topology with the driving potential gives rise to transfer via exotic topological effects, giving extremely fine control over process of long-range doublon transfer.

## Results

### Doublon dynamics

In the limit of strong interactions, fermions on a lattice can pair to form stable bound states known as “doublons”, even if the interaction is repulsive. This effect is a consequence of the discretization of space; the kinetic energy of a particle is limited by the width of the energy band, and so if the interaction energy is sufficiently large, the decay of the doublon into free particles is forbidden on energetic grounds. Doublons have been observed in several systems such as ultracold atomic gases^19^ and in organic salts^23^.

**Figure 1:**
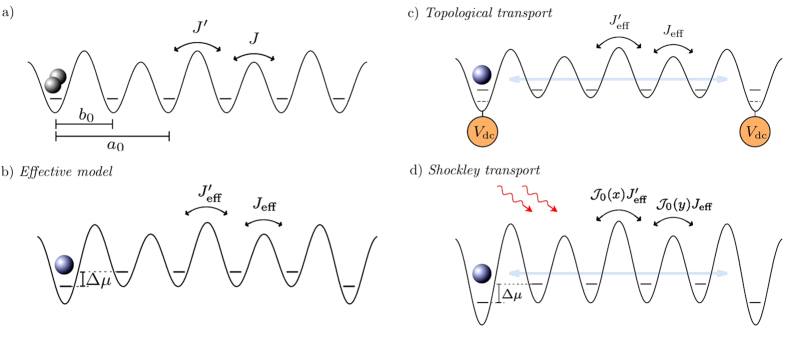
Schematic representations. (**a**) The full Hamiltonian (1). A chain of M dimers characterized by two hopping rates, *J* and *J*′, the lattice constant, *a*_0_, and the intra-dimer distance, *b*_0_. The other important parameter of the model is the interaction strength, *U*, which needs to be large enough with respect to the hoppings for doublons to form. (**b**) Scheme displaying the main features of the effective model Hamiltonian (2). We consider the doublon as a single quasiparticle which moves through the lattice with hoppings 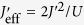 and *J*_eff_ = 2*J*^2^/*U*. In a finite system, a chemical potential difference arises between the endpoints and the rest of the lattice sites. (**c**) Scheme showing how to produce topological long-range transfer of doublons. A gate potential at the terminating sites of the chain is needed to restore the lattice periodicity. (**d**) Scheme showing the Shockley long-range transfer of doublons. The ac-field renormalizes the doublon hoppings to 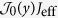 and 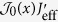, where *x* = 2*Eb*_0_/*ω* and *y* = 2*E*(*a*_0_ − *b*_0_)/*ω*, leaving the chemical potential unaffected.

The system we have studied can be modelled by a SSH-Hubbard Hamiltonian:


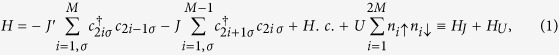


where 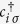 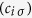 is the standard creation (annihilation) operator for a fermion of spin *σ* on site *i*, and 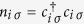 is the number operator. The hopping Hamiltonian *H*_*J*_ is parameterized by the two hopping parameters *J* and *J*′ which describe the dimer structure of the lattice (shown schematically in Fig. 1a), while *H*_*U*_ accounts for the interactions between particles by a Hubbard-*U* term.

We study the two fermion case, the smallest number of fermions that can form a doublon, and restrict ourselves to the singlet subspace (one up-spin and one down-spin). In order to obtain an effective Hamiltonian that accurately models the dynamics of doublons, we can perform a unitary transformation perturbatively in powers of *J*/*U* and *J*′/*U*^24^ (see Methods). Assuming we only have one doublon in the system, we can neglect interaction terms between doublons and the hopping processes of single particles, to arrive at


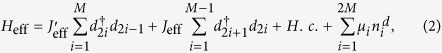



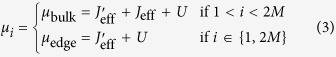


**Figure 2:**
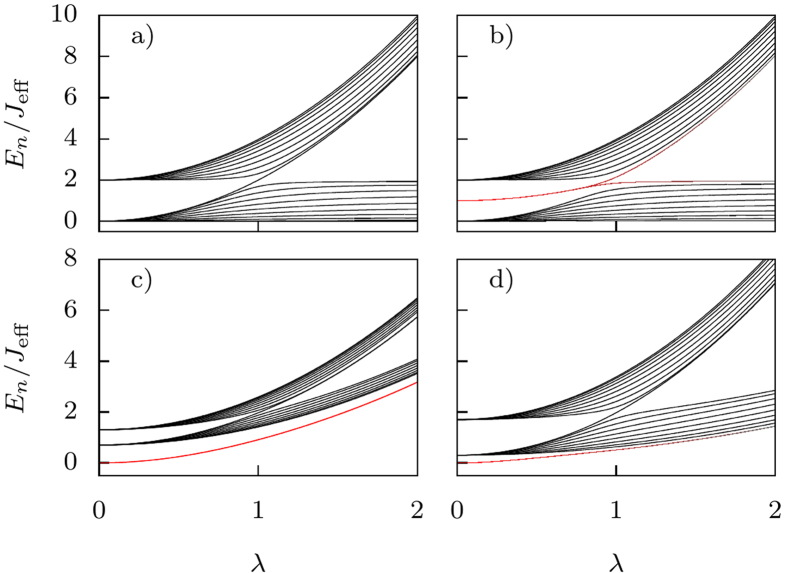
Energy levels of a 10 dimer chain, *b*_0_ = *a*_0_/2, the energy is measured in units of *J*_eff_. (**a**) Without a gate potential, Δ*μ* = −2*J*^2^/*U*, there are no states outside the bulk bands, and therefore no edge states for any value of *λ*. The interaction destroys the edge states as long as the system is in the strongly-interacting regime and no ac field is applied. (**b**) Adding a gate potential to compensate for Δ*μ*, so that Δ*μ* + *μ*_gate_ = 0. The two states with energies in the gap (red lines) for *λ* < *λ*_*c*_ are the edge states predicted by the topology of the system. For *λ* ≥ *λ*_*c*_ the gap closes, and the system becomes topologically trivial. (**c**) Driven by an ac-field with intensity and frequency such that 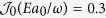, two localized states separate from the bottom of the lowest band (red lines). We can see how the ac field renormalizes the hoppings, making the bands narrower and increasing their separation from the Shockley states. (**d**) Same case with 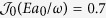.

Here 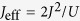 and 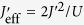. 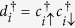, (*d*_*i*_) is the creation (annihilation) operator for a doublon on site *i*, and 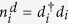 is the doublon number operator. In the effective model (2), the doublon hopping rates are smaller than the original ones and positive regardless of the sign of *J* or *J*′ (see Fig. 1b). Unexpectedly, this transformation also gives rise to a chemical potential term, *μ*_*i*_, which depends on the number of neighbors of site *i*. While all sites in the bulk of the chain have two neighbors, the two end-sites have only one and thus experience a different value of *μ*_*i*_, which breaks the lattice periodicity. The difference in chemical potential is given by 
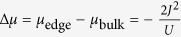
. In Fig. 2a we show the energy spectrum for a chain of 10 dimers, where we can clearly see that even when the system is topologically non-trivial (*λ* < *λ*_*c*_, see below), no edge states are visible. This is a consequence of this finite-size effect; the alteration in chemical potential at the ends of the lattice causes the edge edge states’ energies to enter the bulk bands. As a consequence the system does not support edge states for doublons. This corroborates the result that interactions destroy topological transfer.

### Topological transfer

Analogously to the non-interacting case, the topology in the present case is determined by the ratio between the effective hoppings, given by 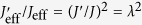. For an infinite chain, the system is in the topologically non-trivial phase when *λ* < *λ*_*c*_ = 1; for the finite case of *M* dimers the critical value of the ratio is given by 
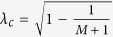
16.

To obtain topological transfer for doublons, we must restore the lattice periodicity by adding a gate voltage, *μ*_gate_, to the edge sites to compensate for the difference in chemical potential, such that Δ*μ* + *μ*_gate_ = 0. In this way we recover edge states for a chain with doublons. We show the result in Fig. 2b, and we can indeed see that the two edge states lie between the bulk bands for *λ* < *λ*_*c*_.

**Figure 3:**
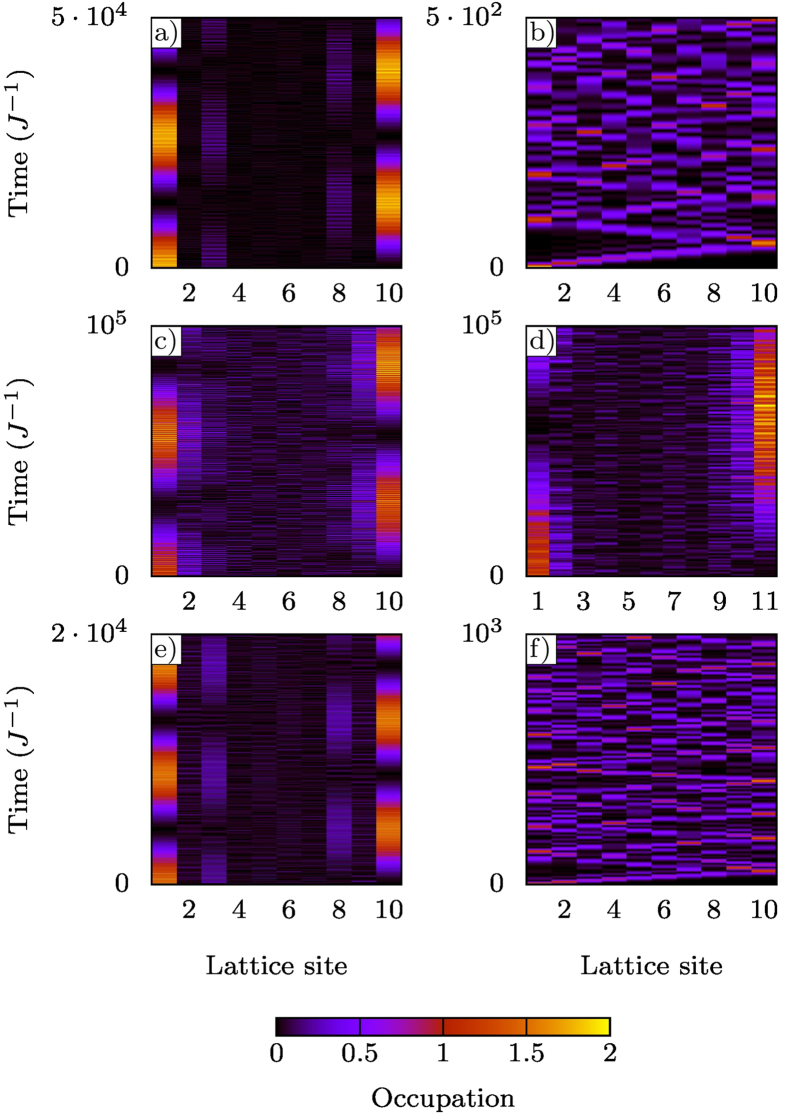
Time evolution of the site occupation. In all cases *U* = 16*J* and the initial condition consists of two fermions in a singlet state occupying the first site of the chain. The simulations are for a chain containing 5 dimers except for (**d**). *Topological transfer*: (**a**) Chain with compensating gate potentials at the edge-sites and *λ* = 0.5 < *λ*_*c*_ (topological regime). The doublon oscillates from one edge to the other without occupying intermediate sites, giving an example of long-range topological transfer in an interacting system. (**b**) As before, but with *λ* = 1 > *λ*_*c*_ (trivial regime). The doublon now simply spreads over the entire lattice. *Shockley transfer*: (**c**) Chain driven by an ac field with parameters *λ* = 1, *b*_0_ = *a*_0_/2, *E*/*ω* = 1.6/*a*_0_ and *ω* = 2*J*. By using the ac field to renormalise the effective hoppings, we can obtain long-range transfer without compensating the chemical potentials of the edge points. (**d**) AC driven chain with same parameters, but an odd number of sites. Long-range transfer is mediated by the Shockley mechanism (no topological transfer would be possible in this case). *AC induced topological transfer*: (**e**) AC driven system with compensating gate potentials, parameters are *λ* = 1.2, *b*_0_ = 0.6*a*_0_, 2*E*/*ω* = 3.6/*a*_0_ and *ω* = 2*J* (topological regime, red square in Fig. 5b). Long range transfer occurs unlike in the undriven system (*λ* = 1.2). (**f**) As in (**e**) but with 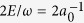 (trivial regime, green dot in Fig. 5b). As expected, no long range transfer occurs.

We show examples of the dynamics in Fig. 3a,b; in the topological regime the doublon oscillates between the two edge-sites without passing through intermediate sites, whereas in the trivial regime the doublon simply spreads over the entire lattice. Interestingly, due to the sublattice symmetry of the system, when the number of sites is odd, there is one and only one edge state in the chain, localized on one end or the other depending on whether *λ* < *λ*_*c*_ or *λ* > *λ*_*c*_ (Supplementary Material). Thus, there is *no* long-range doublon transfer for systems with an *odd* number of sites (half-integer number of dimers).

### Shockley transfer

The effective Hamiltonian for doublons (2) contains, as we discussed above, a site-dependent chemical potential which breaks translational symmetry. This produces Shockley-like surface states^25^ if the hopping rates *J*_eff_ and 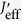 are smaller than 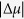. Usually this is not the case, however there is an efficient way to induce such states by driving the system with a high-frequency ac-field. The ac-field renormalizes the hoppings^22^ which become smaller than in the undriven case. This cannot be achieved, for example, by simply reducing the hoppings *J* and *J*′ by hand, since this will also affect the effective chemical potential which still will be of the same order of *J*_eff_ and 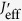. To model the driven system we add a periodically oscillating potential that rises linearly along the lattice


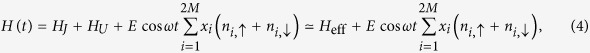


where *E* and *ω* are the amplitude and frequency of the driving, and *x*_*i*_ is the spatial coordinate along the chain. Since the Hamiltonian (4) is periodic in time, *H*(*t*) = *H*(*t* + *T*), we can apply Floquet theory and seek solutions of the Schrödinger equation of the form 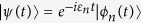, where *ε*_*n*_ are the so called Floquet quasienergies, and 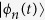 are a set of *T*-periodic functions termed Floquet states. Quasienergies play the same role in the time evolution of the system as conventional energies do for a static Hamiltonian. In the strongly interacting regime, a perturbative calculation shows that the hopping terms are renormalized by the zeroth Bessel function (see Methods), 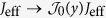, and 
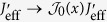
, where *y* = 2*E*(*a*_0_ − *b*_0_)/*ω* and *x* = 2*Eb*_0_/*ω*^17^,^20^. We show the effect of this renormalization in Fig. 2c; as the effective tunneling reduces in magnitude the bulk bands becomes narrower, and the Shockley states are pulled further out of them. The factor of 2 in the argument of 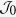 comes from the doublon’s twofold electric charge. The geometry, which so far has not played any role, now becomes important in this renormalization of the hoppings. The simplest case is for *b*_0_ = *a*_0_/2, in which both hoppings are renormalized by the same factor 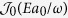. An important point is that the on-site effective chemical potential, being a local operator, commutes with the periodic driving potential, and so is not renormalized. This is the critical reason for using a periodic driving to modify the tunneling; it renormalizes the values of *J*_eff_ while keeping the chemical potential unchanged.

We show in Fig. 2c,d how varying the hopping rates has the effect of pulling two energies out of the bulk bands, inducing the presence of localized states at the edges. These edge states occur in pairs and so also form a non-local two level system^25^. Nevertheless they can be affected by local perturbations and so unlike the previous case, are topologically unprotected^26^. From the stationary eigenstates of Hamiltonian (2) with renormalized hoppings, we can define a quantity, 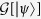, that measures the density correlation between the end-sites for a given eigenstate, 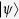,


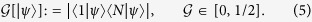


**Figure 4:**
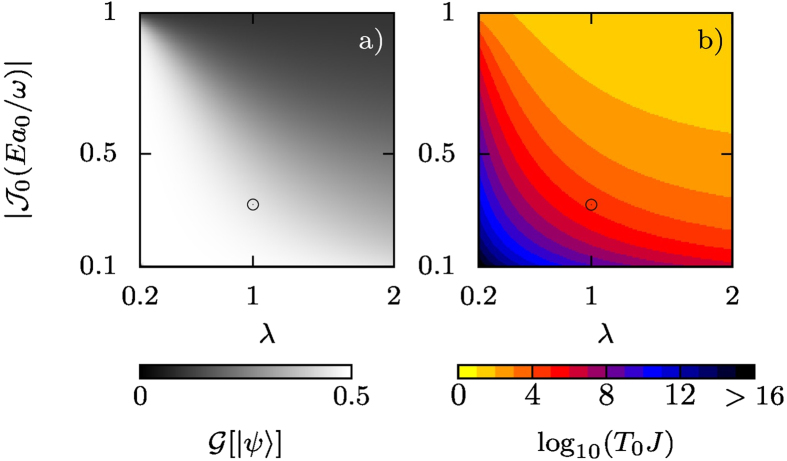
Characterizing Shockley transfer. (**a**) Correlation, 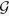, between the edge occupancy of the Shockley-like surface states in an ac-driven chain containing 5 dimers. We have considered the case *b*_0_ = *a*_0_/2. The long-range transfer occurs in the pale region (lower-left) of the parameter space. (**b**) Transfer time, *T*_0_, computed as *π*/Δ*ε*, where Δ*ε* is the energy difference between the two edge states. *T*_0_ tends to infinity as 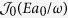 or *λ* go to zero. The black circles correspond to the parameters of the time evolution shown in Fig. 3c; the transfer time, *T*_0_ ~ 10^4^ *J*^−1^, is correctly reproduced. As can be seen, a slight change in the field parameters can change the transfer time by several orders of magnitude.

Here 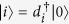 is the basis of localized doublon states. If the total occupancy at the ends, 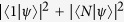, is a constant then 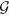 is maximum when 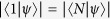. In addition, the energy difference between the two edge states tells us how fast the doublon transfer time is, *T*_0_ = *π*/Δ*ε*. We can see in Fig. 4a that when the values of the hoppings are reduced by the ac field, the two lowest energy eigenstates of Hamiltonian (2) become more localized at the edges. Smaller values of *λ* favor localization as well. This produces cleaner dynamics with less unwanted occupancy of the intermediate sites of the chain. On the other hand, we can see in Fig. 4b that the transfer time rapidly increases, soon becoming too large to observe in simulations or in experiment. At larger values of the hoppings the edge states enter the bulk bands, as can be seen in Fig. 2d close to *λ* = 1, and the long-range transfer of doublons is suppressed.

We show examples of the dynamics for a periodically-driven system in Fig. 3c,d. Since the origin of the edge states is not topological, long-range transfer can occur via this mechanism for chains even with an odd number of sites, as seen in Fig. 3d.

### AC induced topological transfer

If we combine both methods, adding a gate potential at the ends *and* driving the system with an ac-field, it is possible to bring the system into exotic topological phases. The effective Hamiltonian is simply given by (11) without the chemical potential term.

There is a close connection between the correlation of the edge-occupancy, 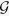, for those states which close the gap, and the Zak phase, 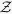
27. This is the topological invariant that classifies 1D Hamiltonians with time reversal, particle-hole and chiral symmetry. The Zak phase has already been calculated for a driven dimer chain without interaction^17^, and it is straightforward to extend it to our effective model for doublons


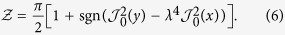


**Figure 5:**
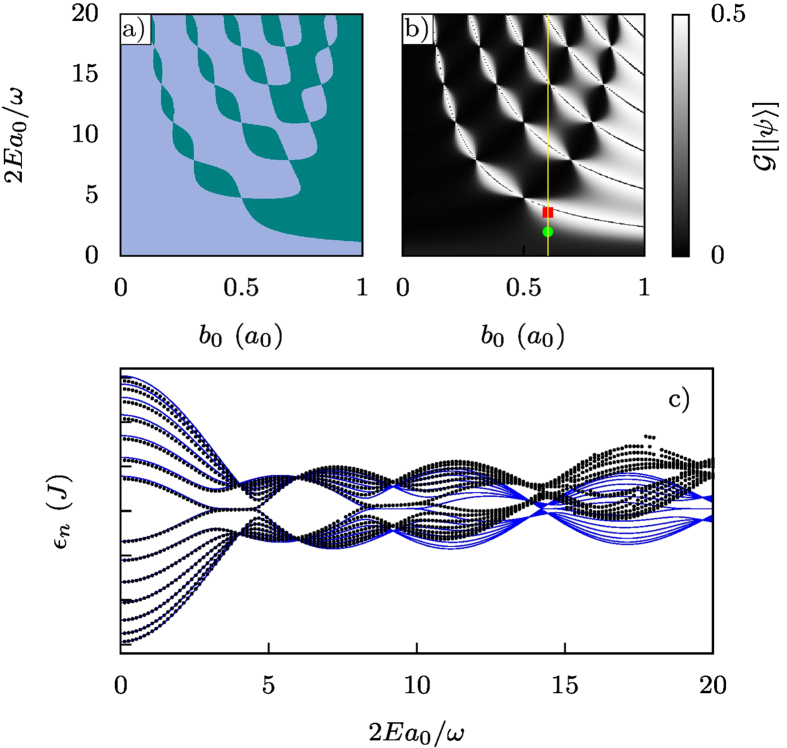
Exotic topological transfer. (**a**) Plot of 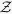 (Eq. 6) for *λ* = 1.2; green regions are those in which the system is in the topologically non-trivial phase 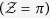. (**b**) Plot of 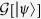 computed for a chain containing 7 dimers with the same *λ* as in (**a**). The black lines that cross the white regions correspond to the zeros of 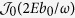. At those points in parameter space, the different sites of the chain are uncoupled; the system is thus degenerate and 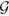 is not a well-defined quantity. The yellow line marks the cross-section through parameters space used in the quasienergy plot. The red square and green dot mark the parameters of the system for the time evolutions shown in Fig. 3e,f respectively. (**c**) Quasienergies as a function of the driving amplitude *E*. The other parameters are set to *U* = 16*J, b*_0_ = 0.6*a*_0_ and *ω* = 2*J*. The edge states appear as predicted by the phase diagram, and correspond to the highest values of 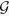. For large values of the ac-field intensity, the doublon states begin to couple with the other states of the system and the exact quasienergies (black dots) diverge from those predicted by the effective model (blue lines).

The argument of the Bessel functions is twice that for a non-interacting system, and the factor *λ*^4^ comes from the squared ratio between the effective doublon hoppings. In Fig. 5a,b we compare the phase diagram obtained by plotting (6) and the result obtained by computing 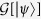. We can see that the agreement is excellent, indicating that the Zak phase can be directly measured from the density correlation function. In Fig. 5c we show the quasienergy spectrum for *b*_0_ = 0.6*a*_0_, to make a cross-section through the parameter space. It can clearly be seen that when the system is topologically non-trivial, corresponding to 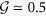, a pair of edge states emerges from the bulk bands and enters the gap. When the system is topologically trivial they then reenter the bulk again. The dots in Fig. 5c were obtained from the diagonalization of the unitary time-evolution operator for one period of the full original Hamiltonian (4) with an added gate potential, making no approximations. The agreement between these quasienergies, and those calculated from the effective model (11) is extremely good for driving parameters 2*Ea*_0_/*ω* ≤ 10, indicating that our approximation schemes are valid. For larger values of the driving parameters our effective model still captures the behaviour of the quasienergies, but small deviations begin to appear as the doublon states begin to couple with other states of the system.

In Fig. 3e,f we show two examples of the dynamics corresponding to the two points marked in Fig. 5b. When the system is topologically non-trivial (red square) the system exhibits long-range doublon transfer as expected. In the topologically trivial regime (green dot), however, this does not occur, and the doublon instead propagates throughout the whole lattice.

## Discussion

We have derived an effective Hamiltonian for two particles in a quantum dimer chain that bind together via a repulsive interaction. Interestingly this binding produces an effective surface potential, different from that of the bulk. In general this surface potential prevents topological transfer of particles, but by adding local gate potentials to compensate for it, topological transfer can be recovered. We have also shown that by adding a periodic driving potential to renormalize the hoppings, while leaving the surface potential unchanged, we can produce long-range transfer via Shockley states. This transfer is, however, not topologically protected. Finally, by combining topological transfer with an ac driving field, we can obtain a rich topological phase diagram, in which long-range transfer occurs when the Zak phase is non-zero.

It is natural to ask how this long-range transfer phenomena depend on the total number of dimers forming the chain. The transfer time is essentially the inverse of the energy difference in the two-level system formed by the hybridization of the edge states. This energy difference is related to the overlap between the edge states, which are solutions that decay exponentially from the surface of the lattice. Thus we can conclude that the transfer time increases exponentially when increasing the size of the chain. Another fact which affects the transfer time is the dependence of 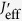 and *J*_eff_ on *U*. Increasing the interaction strength has the effect of slowing down the dynamics.

Ultracold atoms confined in optical lattice potential are extremely clean and only slightly affected by decoherence. In units of the tunneling time, doublon life time in a three dimensional optical lattice, has been found to depend exponentially on the ratio of the on-site interaction to the kinetic energy^28^. This is not in general the case for electron transfer in semiconductor nanostructures where hyperfine or spin-orbit orbit interactions induce decoherence, which strongly depends on the material. However, since we deal with doublons, forming a singlet state, spin relaxation and decoherence is suppressed by the energy difference between the intradot singlet and excited triplet states.

In summary, we propose three ways for long-range transfer of strongly-interacting particles, all mediated by edge states. In the first case, non-trivial topological edge states are required. In the second, long-range transport is mediated by Shockley states induced by ac driving. Finally, combining both topology and driving allows us to tune the range of parameters where long-range transfer is achieved. Our proposal could be experimentally confirmed both in cold atoms and in semiconductor quantum dot arrays. In these last systems either charge detection by means of a quantum detector, such as a quantum point contact or an additional quantum dot, or transport measurements are within experimental reach.

Our results open new avenues to achieve direct transfer of interacting particles between distant sites, an important issue for quantum information architectures.

## Methods

### Effective Hamiltonian for doublons

The energies of a one-dimensional lattice form a Bloch band with a width of 2*J*, and thus the maximum kinetic energy carried by two free particles is 4*J*. If the particles are initially prepared in a state with a potential energy much greater than 4*J*, the initial state then cannot decay without the mediation of dissipative processes. We consider the regime where for doublons to split is energetically unfavorable, i.e. 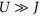, *J*′. Following the article by Hofmann and Potthoff 24 we obtain an effective Hamiltonian just for the doublons in a dimer chain by means of a Schrieffer-Wolff (SW) transformation, projecting out the single-occupancy states. This transformation is performed perturbatively in powers of *J*/*U* and *J*′/*U* and up to second order gives rise to the effective Hamiltonian (2) where the hoppings *J* and *J*′ become renormalized by the interaction.

### AC driven Hamiltonian: hopping renormalization

If one wants to deal with interactions between particles as well as interactions with an external driving, and treat both on an equal footing, a more elaborate procedure than before is necessary. For a time-periodic Hamiltonian *H*(*t* + *T*) = *H*(*t*) with *T* = 2*π*/*ω*, the Floquet theorem states that the time evolution operator *U*(*t*_2_, *t*_1_) can be written as:


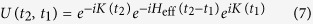


with a time-independent effective Hamiltonian, *H*_eff_ governing the slow dynamics and a *T*-periodic operator *K*(*t*) that accounts for the fast dynamics; exp(−*iK*(*t*)) is also termed the *micromotion operator*. In the high-frequency limit, by which we mean 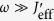 and *J*_eff_, these operators can be expanded in powers of 1/*ω*:


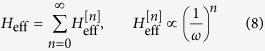


For a detailed description of the high frequency expansion (HFE) method see^29^,^30^,^31^. Now we express our periodic Hamiltonian (4) in the rotating frame with respect to both the interaction and the ac field^32^:


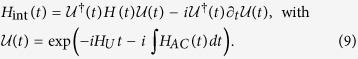


Here 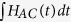 is just the antiderivative of the operator:


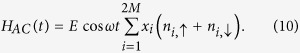


To derive the effective Hamiltonian, we perform the HFE of *H*_int_(*t*) up to first order in 1/*ω*, see Supplementary Material. It can be seen that in the limit 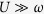 the result is the same as the one obtained by first performing the SW transformation and then the hoppings renormalization. Conversely, in the limit 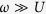 the result is consistent with first doing the high-frequency hopping renormalization and then the SW transformation. This coincidence can be understood since the great difference between *U* and *ω*, permits the separation of the different time scales associated with each energy in the HFE. The effective Hamiltonians in the two different regimes are:


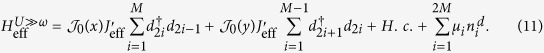



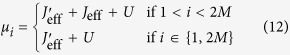



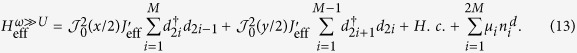



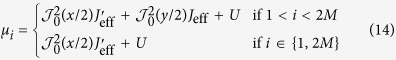


We emphasize that only in the regime 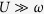 the effect of the ac field is to renormalize the hopping parameters but not the effective chemical potential. This is a nontrivial point, key to the understanding of the Shockley transfer phenomenon.

## Additional Information

**How to cite this article**: Bello, M. *et al*. Long-range doublon transfer in a dimer chain induced by topology and ac fields. *Sci. Rep.* **6**, 22562; doi: 10.1038/srep22562 (2016).

## Supplementary Material

Supplementary Information
