# Supplementary material for: Long-range doublon transfer in a dimer chain induced by topology and ac fields
Source: Sci Rep. 2016 Mar 2;6:22562. doi: 10.1038/srep22562 (PMC4773839; doi:10.1038/srep22562)
Supplement: Supplementary Information [file srep22562-s1.pdf]

Long-range doublon transfer in a dimer chain induced by topology and ac fields

M. Bello,[1] C.E. Creffield,[2] and G. Platero[1]
[1]*Instituto de Ciencias de Materiales, CSIC, Cantoblanco, E-28049, Madrid, Spain*
[2]*Departamento de Física de Materiales, Universidad Complutense de Madrid, E-28040, Madrid, Spain*
(Dated: January 7, 2016)

SUPPLEMENTARY INFORMATION

Non-interacting system. Topological properties of the SSH model.

The tight-binding Hamiltonian for a one-dimensional chain of M dimers has the following form

$$H = J' \sum_{i=1}^{M} c_{2i}^{\dagger} c_{2i-1} + J \sum_{i=1}^{M-1} c_{2i+1}^{\dagger} c_{2i} + H.c. \ , \tag{1}$$

where J, J' are the hopping parameters, and $c_i^{\dagger}$ (c_i) is the creation (annihilation) operator for a fermion of spin σ on site i. As shown in Fig.1, the unit cell is composed of two atoms, A and B, which correspond to sites with odd and even indices respectively. We will use $\{|i\rangle\}_{i=1..N}$ to denote the localized orbitals, $c_i^{\dagger}|0\rangle = |i\rangle$.

Solving the eigenvalue equation, one finds that the energies of a finite chain lie in the bulk bands, with the possible exception of two energies close to zero which correspond to edge states (see Figs. 2,3). The presence or absence of edge states is determined by the topology of the system. For 1D lattice systems, a topological classification can be made according to the value of the so-called Zak phase [1, 2]. If $|u_k\rangle$ is a cell-periodic Bloch function with quasi-momentum k then

$$Z = \oint \langle u_k | i\partial_k | u_k \rangle dk$$

i. e. the Zak phase is obtained from the integration of the Berry connection over the first Brillouin zone, and is a topological invariant. For the dimer chain, this invariant depends on the value of the parameter $\lambda = J'/J$. For an infinite chain, the system only supports edge states [1, 2] if $\lambda < 1$. In that case the system is said to be in the topological phase. Otherwise, the system is in the non-topological, or trivial, phase. For a finite chain the value of λ at which the transition takes place depends on the number of dimers in the chain M, $\lambda_c = \sqrt{1 - 1/(M+1)}$.

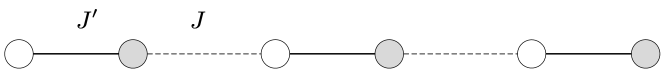

FIG. 1: Scheme of a dimer chain.

The dimer chain consists of two sub-lattices connected by hopping, and so has sub-lattice symmetry. Consequently for any positive eigenvalue of the Hamiltonian (1) there exists a negative eigenvalue with the same absolute value, as we can see in Fig. 2. However, if the chain contains an odd number of sites (a half-integer number of dimers) there is only one edge state, with energy equal to 0. For $\lambda < \lambda_c$ this edge state is localized on the first site of the lattice, whereas for $\lambda > \lambda_c$ it is localized on the last site, as shown in Fig.4.

Long-range particle transfer.

Let us consider a finite dimer chain with an even number of sites. In the topological regime, where there are two edge states $|e_+\rangle$, $|e_-\rangle$, with energies E_+, E_-, one can consider

$$|1\rangle \simeq \frac{1}{\sqrt{2}}(|e_+\rangle + |e_-\rangle), \quad |N\rangle \simeq \frac{1}{\sqrt{2}}(|e_+\rangle - |e_-\rangle)$$

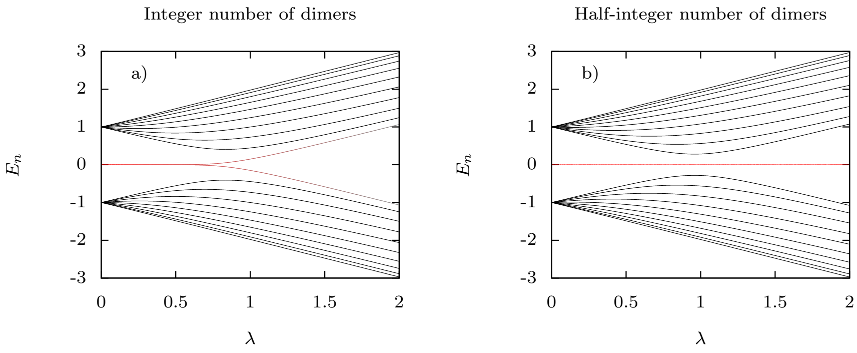

FIG. 2: a) Energy spectrum of the SSH Hamiltonian for a finite chain containing 10 dimers. The energies corresponding to edge states (in red) enter the bulk bands for $\lambda > 1 - 1/11 \simeq 0.91$ and the system no longer supports them. b) The energies for a chain consisting on 10 and a half dimers. As can be seen, now there is always an edge state. This is a consequence of the sub-lattice symmetry.

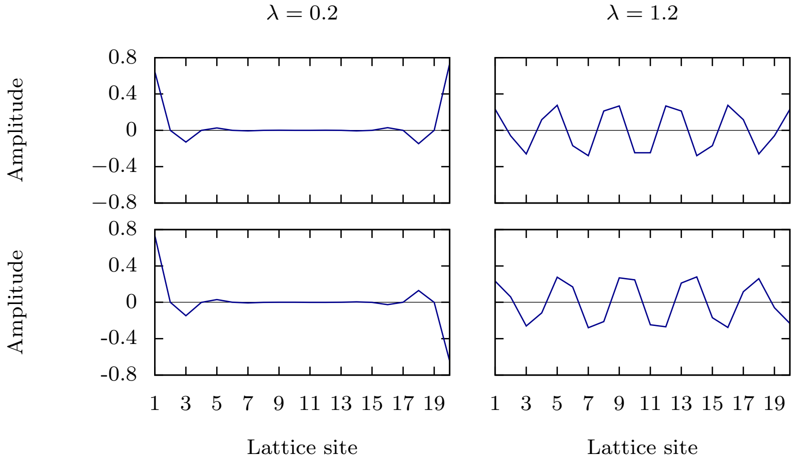

FIG. 3: Plot of the eigenstates for a chain containing 10 dimers, corresponding to the eigenvalues shown in red in Fig. 2 a). Left: the system is in the topological phase and the states are localized at the end sites of the chain. These figures show that the two edge states have different symmetry. Right: the system is in the trivial phase and therefore does not support edge states.

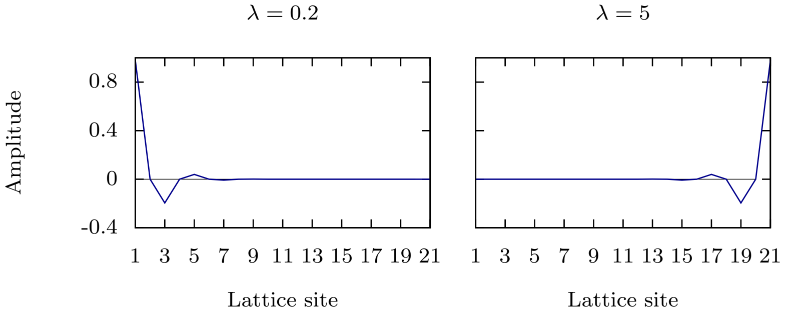

FIG. 4: Plot of the eigenstate for a chain with an odd number of sites (21), corresponding to the eigenvalue in red in Fig. 2b), for $\lambda = 0.2$ (left) and $\lambda = 5$ (right). In each case there is just one edge state with weight just at one edge, which changes from left to right as a function of λ

If the initial condition is $|\psi(t=0)\rangle = |1\rangle$, the state evolves in time as

$$|\psi(t)\rangle \simeq \frac{1}{\sqrt{2}}\left(e^{-i\frac{E_+}{\hbar}t}|e_+\rangle + e^{-i\frac{E_-}{\hbar}t}|e_-\rangle\right)$$

The mean occupation on the edges of the chain is therefore given by

$$\begin{aligned}\langle n_1\rangle &= |\langle 1|\psi\rangle|^2 = \frac{1}{4}\left|e^{-i\frac{E_+}{\hbar}t} + e^{-i\frac{E_-}{\hbar}t}\right|^2 = \frac{1}{2}(1+\cos(\omega_0 t)), \quad \text{with } \omega_0 = \frac{E_+ - E_-}{\hbar} \\ \langle n_N\rangle &= |\langle N|\psi\rangle|^2 = \frac{1}{2}(1-\cos(\omega_0 t))\ .\end{aligned} \tag{2}$$

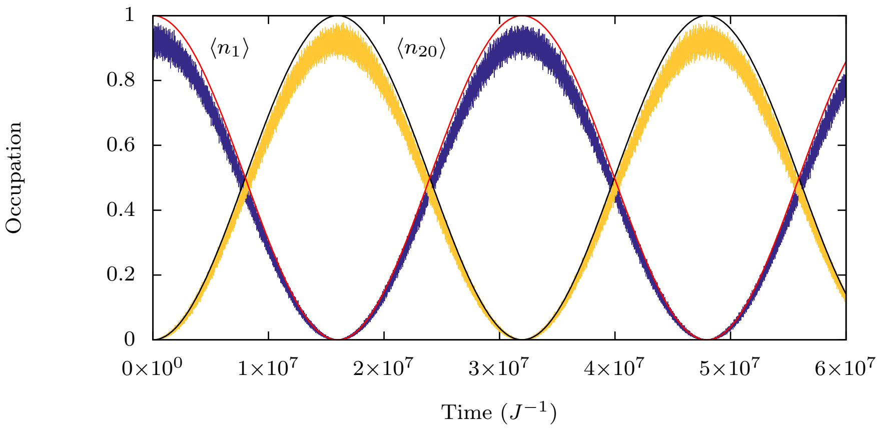

FIG. 5: Time evolution of a system consisting of 10 dimers. The initial condition is the particle being on site 1, i.e. $|\psi(0)\rangle = |1\rangle$. In blue and yellow are the occupations at the two edges of the chain: $n = 1$ and $n = 20$ respectively. In black and red are the approximate occupations given by Eq. (2).

We plot this behavior in Fig. 5, and see that this approximation gives an excellent description of the transfer dynamics. This approximation improves as the edge states becomes more localized at the edges of the chain. In the non-topological regime, however, for the same initial condition, the state will in general be a superposition of more eigenstates and the particle will spread over the entire lattice, see Fig. 6 (center).

Interacting particles: doublon formation.

In the following we will consider two interacting fermions in the singlet subspace. Usually, the interaction destroys the charge oscillation between edges described in the previous section, as shown in Fig 8. When this interaction is large enough, however, the two particles bind together forming what is called a doublon, whose dynamics can be modeled by an effective Hamiltonian (Eq. 2 in the main article). In Fig. 7, we compare the energy spectrum obtained from the original two-particle Hubbard Hamiltonian, and that obtained from the effective doublon Hamiltonian, as a function of the interaction strength, U. The agreement is clearly excellent for $U \geq 4J$.

If one compares the spectral density of a non-interacting dimer array in the topological regime (Fig. 9 left) with that of the effective Hamiltonian for interacting particles (Fig. 9 right), one can see that the inhomogeneity in the system due to the effective chemical potential induced by the interaction removes the edge states. This is why the long-range transfer of doublons does not occur naturally in the dimer chain unless a perturbation is added to the system, such as a gate potential, in order to recover the symmetry.

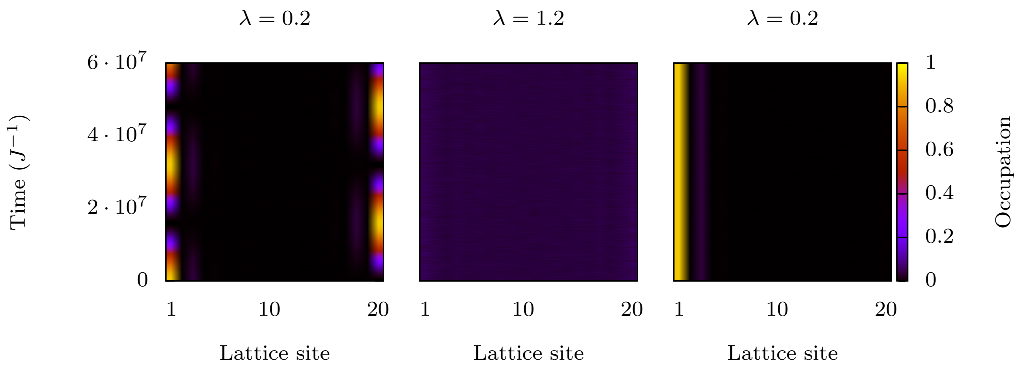

FIG. 6: Time evolution of the occupation in a chain containing 10 dimers, $U = 0$. The initial condition corresponds to a particle being localized on site 1, i.e. $|\psi(0)\rangle = |1\rangle$. On the left, the system in the topological regime, the particle oscillates between both ends of the chain barely occupying the middle sites (long-range transfer). On the center, the system is in the trivial regime. The particle spreads over the entire lattice and the occupation looks practically homogeneous over the lattice. On the right, time evolution of the occupation for a chain containing 10 and a half dimers in the topological regime. Now the particle remains at the initial position at all times; in this case $|1\rangle$ is approximately an eigenstate of the system and is therefore stationary.

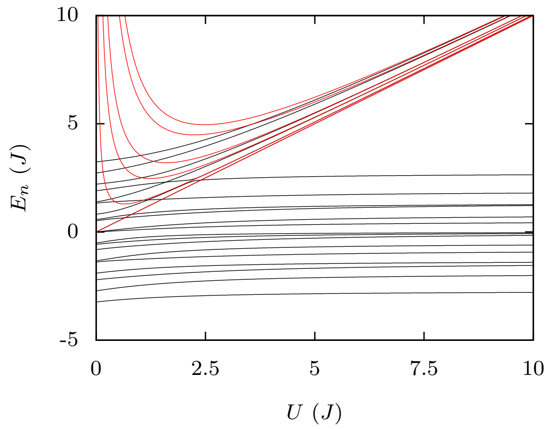

FIG. 7: Eigenvalues computed for a three dimer chain with $\lambda = 0.8$. Already for $U \geq 4J$ we find very good agreement between the excited state energies of the Hubbard Hamiltonian (black lines), which depend linearly on U, and those given by the effective model (red lines). The rest of states are not included in the effective model, they correspond to states where fermions are in different lattice sites.

Effective Hamiltonian for doublons in the presence of an ac field

In this section, we derive the effective Hamiltonian for doublons in a dimer chain coupled to an external periodic driving. The Hamiltonian reads:

$$\begin{aligned} H(t) &= -\sum_{\langle i,j\rangle,\sigma} J_{ij} c^\dagger_{i\sigma} c_{j\sigma} + U \sum_i n_{i\uparrow} n_{i\downarrow} + \sum_i V_i(t)(n_{i\uparrow} + n_{i\downarrow}) \\ &\equiv H_J + H_U + H_{AC}(t) \ , \end{aligned} \tag{3}$$

where J_{ij} is the hopping rate between neighboring sites i and j, and U is the interaction strength between particles occupying the same lattice site. $c^\dagger_{i\sigma}$ ($c_{i\sigma}$) is the usual fermionic creation (annihilation) operator of one particle with spin σ on site i and $n_{i\sigma} = c^\dagger_{i\sigma} c_{i\sigma}$ is the number operator. The periodic potential, $V_i(t + T) = V_i(t)$, has frequency

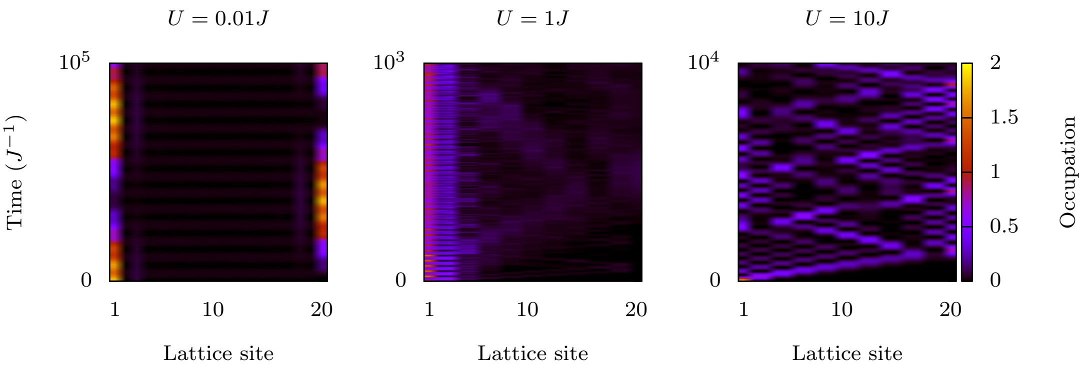

FIG. 8: Time evolution of a system with 10 dimers and $\lambda = 0.2$. The initial condition corresponds to two fermions in a singlet state on the first site of the chain. For small U there is still long-range transfer of particles but they are less localized at the edges than in the non interacting case (Fig. 6 left). As U increases, the two particles spread over the entire lattice.

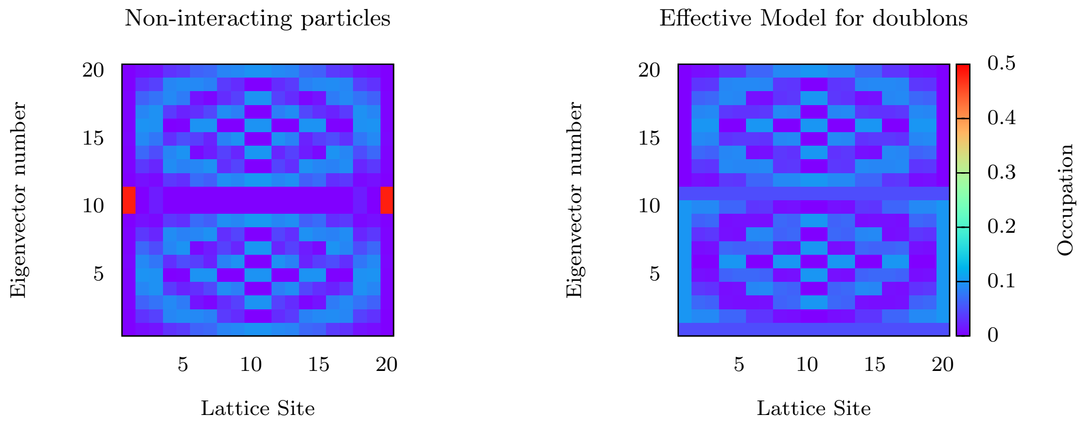

FIG. 9: The squared amplitude of the eigenstates for a system containing 10 dimers. In both plots $\lambda = 0.2$ but only the one on the left, corresponding to non interacting fermions, has edge-states (eigenvalues 10th and 11th).

$\omega = 2\pi/T$ and is the same for all spin species. Going to the rotating frame with respect to both interaction and driving amounts to:

$$H_{\text{int}}(t) = -\sum_{\langle i,j\rangle,\sigma} J_{ij} e^{i\mathbf{A}(t)\mathbf{d}_{ij}} \left[1 - n_{i\bar{\sigma}}(1 - e^{iUt})\right] c^{\dagger}_{i\sigma} c_{j\sigma} \left[1 - n_{j\bar{\sigma}}(1 - e^{-iUt})\right] \tag{4}$$

$$\equiv -\sum_{\langle i,j\rangle,\sigma} J_{ij} e^{i\mathbf{A}(t)\mathbf{d}_{ij}} h^{0}_{ij\sigma} - \sum_{\langle i,j\rangle,\sigma} \left[J_{ij} e^{i\mathbf{A}(t)\mathbf{d}_{ij}} e^{iUt} h^{+}_{ij\sigma} + H.c.\right] \tag{5}$$

Where we have defined:

$$h^{0}_{ij\sigma} \equiv n_{i\bar{\sigma}} c^{\dagger}_{i\sigma} c_{j\sigma} n_{j\bar{\sigma}} + (1 - n_{i\bar{\sigma}}) c^{\dagger}_{i\sigma} c_{j\sigma} (1 - n_{j\bar{\sigma}}) \tag{6}$$

$$h^{+}_{ij\sigma} \equiv n_{i\bar{\sigma}} c^{\dagger}_{i\sigma} c_{j\sigma} (1 - n_{j\bar{\sigma}}) \tag{7}$$

$$(h^{+}_{ij\sigma})^{\dagger} \equiv h^{-}_{ji\sigma} = (1 - n_{j\bar{\sigma}}) c^{\dagger}_{j\sigma} c_{i\sigma} n_{i\bar{\sigma}} \tag{8}$$

Hopping processes described by the operators $h^{+}_{ij\sigma}$ and $h^{-}_{ij\sigma}$ raise and lower by one the total double occupancy of the system respectively, whereas those described by $h^{0}_{ij\sigma}$ leave it invariant. $\mathbf{A}(t)$ is a vector potential from which the ac

field comes, and $\mathbf{d}_{ij}$ is the distance between neighboring sites i and j (note that $\mathbf{d}_{ji} = -\mathbf{d}_{ij}$).

In order to apply the HFE we need to find a common frequency. We will consider first the resonant regime, $U = l\omega$, and then, by means of analytical continuation, obtain the limits $U \gg \omega$ and $U \ll \omega$ [5]. We will approximate $H_{\rm eff}$ only up to first order in $1/\omega$. The different terms in the HFE can be found following several perturbative approaches [6] [7] and are given by the following expressions:

$$H_{\rm eff}^{[0]} = H_{\rm int}^{(0)} \ , \quad H_{\rm eff}^{[1]} = \sum_{q\neq 0} \frac{H_{\rm int}^{(-q)} H_{\rm int}^{(q)}}{q\omega} = \sum_{q=1}^{\infty} \frac{[H_{\rm int}^{(-q)}, H_{\rm int}^{(q)}]}{q\omega} \ , \tag{9}$$

$$\text{with } H_{\rm int}^{(q)} = \frac{1}{T}\int_0^T H_{\rm int}(t) e^{iq\omega t} \mathrm{d}t \tag{10}$$

Now assuming a particular shape of the periodic driving: $V_i(t) = E\cos(\omega t) x_i \Rightarrow A(t) = \frac{E}{\omega}\sin(\omega t)$, the Fourier components of $H_{\rm int}(t)$ are found to be:

$$H_{\rm int}^{(q)} = -\sum_{\langle i,j\rangle,\sigma} J_{ij}\mathcal{J}_q(Ed_{ij}/\omega) h^0_{ij\sigma} + J_{ij}\mathcal{J}_{q+l}(Ed_{ij}/\omega) h^+_{ij\sigma} + J_{ij}\mathcal{J}_{q-l}(Ed_{ij}/\omega) h^-_{ij\sigma} \tag{11}$$

We make some remarks on the different terms of the HFE. First, in the zeroth-order effective Hamiltonian, terms already appear that raise and lower the double occupancy. These are proportional to the Bessel functions of the first kind of order l (the order of resonance) and correspond to the doublon association and dissociation processes assisted by the ac field. However, they can be neglected for small driving amplitudes. As can be seen in Fig. 10, for higher driving amplitudes, the effective model we derive does not give accurate results. Second, in the first-order correction to the effective Hamiltonian, three different kind of terms appear. Those which are a product of $h^0_{ij\sigma}$ and $h^{\pm}_{mn\sigma'}$ do not conserve the total double occupancy. Terms proportional to the product $h^+_{ij\sigma} h^-_{mn\sigma'}$ do preserve the total double occupancy as well as terms proportional to the reversed product of the same operators, but the former act on the doublon subspace whereas the latter act on the single-occupancy subspace. Since we want to describe the dynamics of doublons alone, there are no single-occupancy states in the system, and we have to keep only terms proportional to $h^+_{ij\sigma} h^-_{mn\sigma'}$. Of these, the only non-vanishing terms are:

$$H_{\rm eff}^{[1]} = \frac{1}{\omega}\sum_{q\neq 0}\sum_{\langle i,j\rangle,\sigma} \frac{J_{ij}\mathcal{J}_{-q+l}(Ed_{ij}/\omega) J_{ji}\mathcal{J}_{q-l}(Ed_{ji}/\omega) h^+_{ij\sigma} h^-_{ji\sigma}}{q} + \frac{J_{ij}\mathcal{J}_{-q+l}(Ed_{ij}/\omega) J_{ij}\mathcal{J}_{q-l}(Ed_{ij}/\omega) h^+_{ij\sigma} h^-_{ij\bar\sigma}}{q} \tag{12}$$

Since $J_{ij} = J_{ji}$ and $d_{ij} = -d_{ji}$, the first terms can be written as:

$$\frac{J_{ij}^2}{\omega}\sum_{p\neq -l} \frac{\mathcal{J}_p^2(Ed_{ij}/\omega) h^+_{ij\sigma} h^-_{ji\sigma}}{p+l} = \frac{J_{ij}^2}{U}\sum_{p\neq -l} \frac{\mathcal{J}_p^2(Ed_{ij}/\omega)}{p\omega/U + 1}\left(n_{i\bar\sigma} n_{i\sigma} - n_{i\bar\sigma} n_{i\sigma} n_{j\sigma} n_{j\bar\sigma}\right) \tag{13}$$

And the second term can be written as:

$$\frac{J_{ij}^2}{\omega}\sum_{p\neq -l} \frac{\mathcal{J}_p(Ed_{ij}/\omega)\mathcal{J}_{-p}(Ed_{ij}/\omega) h^+_{ij\sigma} h^-_{ji\sigma}}{p+l} = \frac{J_{ij}^2}{U}\sum_{p\neq -l} \frac{\mathcal{J}_p(Ed_{ij}/\omega)\mathcal{J}_{-p}(Ed_{ij}/\omega)}{p\omega/U + 1} c^\dagger_{i\sigma} c^\dagger_{i\bar\sigma} c_{j\bar\sigma} c_{j\sigma} \tag{14}$$

Strong interacting regime: $U \gg \omega > J_{\rm eff}, J'_{\rm eff}$

In the limit $U \gg \omega$ we can ignore $p\omega/U$ in the denominators of the above expressions. We note that in the analytical continuation of these expressions, the restriction $p \neq -l$ has no meaning. Now using the identities:

$$\sum_{q=-\infty}^{\infty} \mathcal{J}_q^2(\alpha) = 1 \ , \quad \sum_{q=-\infty}^{\infty} \mathcal{J}_q(\alpha)\mathcal{J}_{k-q}(\beta) = \mathcal{J}_k(\alpha+\beta) \ , \tag{15}$$

we arrive at:

$$\boxed{\begin{aligned} &H_{\rm eff}^{U\gg\omega} = \sum_{\langle i,j\rangle} J_{ij}^{\rm eff}\mathcal{J}_0(2Ed_{ij}/\omega) d_i^\dagger d_j + \sum_i \mu_i^{\rm eff} n_i^d - \sum_{\langle i,j\rangle} \frac{2J_{ij}^2}{U} n_i^d n_j^d \\ &J_{ij}^{\rm eff} := 2J_{ij}^2/U \ , \quad \mu_i^{\rm eff} := \sum_j J_{ij}^{\rm eff} \end{aligned}} \tag{16}$$

which has been written as a function of the doublon operators $d_i^\dagger = c_{i\uparrow}^\dagger c_{i\downarrow}^\dagger$ and $n_i^d = d_i^\dagger d_i$. Here J_{ij} is either J or J', and d_{ij} is what we have called b_0 or $(a_0 - b_0)$. The sum in the definition of $\mu_i^{\rm eff}$ is carried over the neighbours of site i. The first term corresponds to the hopping processes of doublons. The second one corresponds to a chemical potential that depends on the specific lattice characteristics, and the last term corresponds to an attractive interaction between neighboring doublons. As we consider just one doublon in our system, we can neglect this term. In order to back up our reasoning, we present in Fig. 10 a comparison between the derived effective Hamiltonian and exact numerical results.

High-frequency regime: $\omega \gg U > J_{\rm eff}, J'_{\rm eff}$

In the limit $\omega \gg U$ all terms in the series are very small except those for $p = 0$ and we can approximate:

$$\boxed{\begin{array}{l} H_{\rm eff}^{\omega\gg U} = \sum_{\langle i,j\rangle} J_{ij}^{\rm eff}\mathcal{J}_0^2(Ed_{ij}/\omega)d_i^\dagger d_j + \sum_i \mu_i^{\rm eff} n_i^d - \sum_{\langle i,j\rangle} J_{ij}^{\rm eff}\mathcal{J}_0^2(Ed_{ij}/\omega)n_i^d n_j^d \\ J_{ij}^{\rm eff} := 2J_{ij}^2/U\ , \quad \mu_i^{\rm eff} := \sum_j J_{ij}^{\rm eff}\mathcal{J}_0^2(Ed_{ij}/\omega) \end{array}} \tag{17}$$

In Fig. 11 we again compare the quasienergy spectrum given by this effective theory with the exact results. It is important to note that now the chemical potential, μ_i, also depends on the parameters of the driving and we do not expect Shockley transfer to occur.

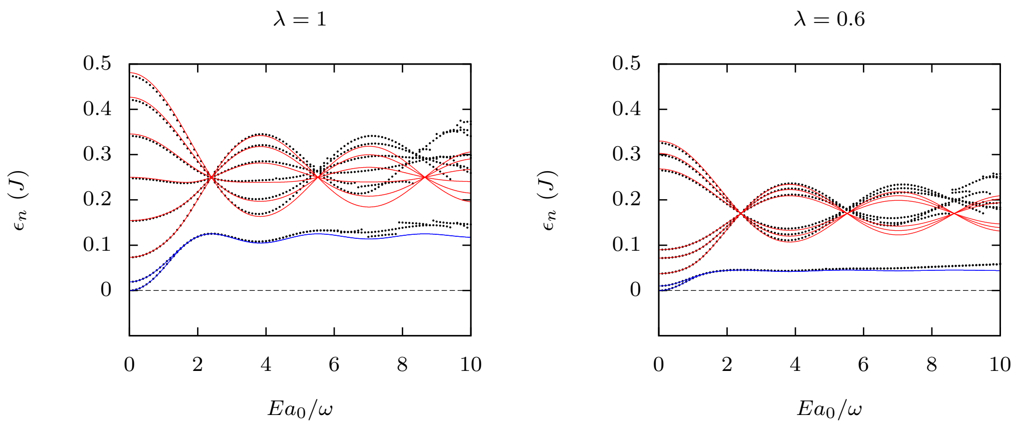

FIG. 10: Quasienergy spectrum as a function of the field amplitude E for a four dimer chain with $b_0 = a_0/2$ in the strongly interacting regime $U = 16J$ and $\omega = 2J$. The dots correspond to the exact quasienergies obtained numerically from the whole Hamiltonian (3), whereas the lines correspond to the eigenvalues of the perturbative Hamiltonian (16). The two lower quasienergies (in blue) correspond to edge states. Left: $\lambda = 1$, i.e. the case of a normal atomic chain; Right: $\lambda = 0.6$ corresponding to a dimer chain.

Shockley transfer. Analytical approximation of the occupancy correlations in an atomic chain.

It turns out that the direct doublon transfer in the case of a chain driven by an ac field does not have a topological origin. Thus it is not surprising that it can occur for values of $\lambda \geq 1$. Let us consider for simplicity the case where $b_0 = a_0/2$. In the limit where $U > \omega \gg J, J'$ the Hamiltonian (16) can be written as

$$H = H^0 + H' \tag{18}$$

$$H^0 := \mu(n_1 + n_N) \tag{19}$$

$$H' := \mathcal{J}_0\left(\tfrac{Ea_0}{\omega}\right)\left[J'_{\rm eff}\sum_{i=1}^{M} d_{2i}^\dagger d_{2i-1} + J_{\rm eff}\sum_{i=1}^{M-1} d_{2i+1}^\dagger d_{2i} + H.c.\right]\ . \tag{20}$$

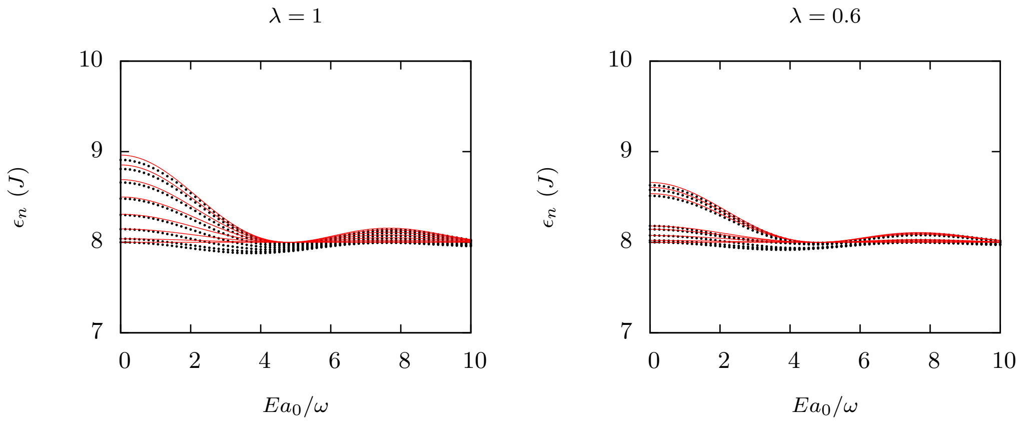

FIG. 11: Quasienergy spectrum as a function of the field amplitude E for a four dimer chain, with $b_0 = a_0/2$. High frequency regime, $\omega > U$ where $U = 8J$ and $\omega = 20J$. The dots correspond to the quasienergies obtained numerically from the full Hamiltonian (3), whereas the lines correspond to the eigenvalues of the effective Hamiltonian (17). Left: normal chain, i.e., $\lambda = 1$; Right: dimer chain, $\lambda = 0.6$. In both cases, there are no edge states. In addition, the dependence on the field amplitude is equivalent to that of a single particle.

Here we have performed an energy shift to bring the chemical potential in every site equal to zero, except for those at the edges of the chain where $\mu = -J_{\rm eff}$. Now, the subspace of states spanned by $|1\rangle$ and $|N\rangle$ is degenerate, with energy $E_D = \mu$. The perturbation does not couple those states at first order, so one needs to go to second order to find the linear combination of states that approximates the eigenstate of the whole Hamiltonian. First we diagonalise the Hamiltonian in the degenerate subspace with energy equal to zero. We are going to consider the case where $\lambda = 1$, which can be analytically treated. In the base of localized orbitals, $\{|i\rangle\}_{i=2..N-1}$, the matrix representation of H is a special case of a tridiagonal Toeplitz symmetric matrix, whose eigenvalues and eigenstates have the following analytical expressions

$$H = \begin{pmatrix} 0 & J_{\rm eff} & & 0 \\ J_{\rm eff} & \ddots & \ddots & \\ & \ddots & & J_{\rm eff} \\ 0 & & J_{\rm eff} & 0 \end{pmatrix} \qquad \begin{array}{l} \epsilon_n = 2J_{\rm eff}\cos\left(\frac{n\pi}{N-1}\right); \quad n = 1,..,N-2 \\ \\ |x_n\rangle = \sum\limits_{m=1}^{N-2}\sqrt{\frac{2}{N-1}}\sin\left(\frac{nm\pi}{N-1}\right)|m+1\rangle; \quad m = 1,..,N-2 \end{array} \tag{21}$$

Up to second order, the perturbation in the subspace spanned by $\{|1\rangle, |N\rangle\}$ is

$$\langle\beta|H^{(2)}|\alpha\rangle = \langle\beta|H'|\alpha\rangle + \sum_n \frac{\langle\beta|H'|x_n\rangle\langle x_n|H'|\alpha\rangle}{\mu - \epsilon_n\mathcal{J}_0\left(\frac{Ea_0}{\omega}\right)} \, . \tag{22}$$

Here $|\alpha\rangle, |\beta\rangle \in \{|1\rangle, |N\rangle\}$ and $\langle\beta|H'|\alpha\rangle = 0$. After some algebra we find

$$H^{(2)} = \begin{pmatrix} a & b \\ b & a \end{pmatrix} \qquad \text{where} \qquad \begin{array}{l} a = \mathcal{J}_0\left(\frac{Ea_0}{\omega}\right)\sum\limits_{n=1}^{N-2}\frac{2\sin^2\left(\frac{n\pi}{N-1}\right)/(N-1)}{\mu-\epsilon_n\mathcal{J}_0\left(\frac{Ea_0}{\omega}\right)} \\ \\ b = \mathcal{J}_0\left(\frac{Ea_0}{\omega}\right)\sum\limits_{n=1}^{N-2}\frac{2\sin\left(\frac{n\pi}{N-1}\right)\sin\left(\frac{n(N-2)\pi}{N-1}\right)/(N-1)}{\mu-\epsilon_n\mathcal{J}_0\left(\frac{Ea_0}{\omega}\right)} \end{array} \tag{23}$$

which has eigenvalues and eigenvectors

$$\epsilon_\pm = a \pm |b| \qquad |\psi^0_\pm\rangle = \frac{1}{\sqrt{2}}[|1\rangle \pm |N\rangle]$$

In the limit where $\mathcal{J}_0(x) \ll 1$, for $b \neq 0$ and $\lambda = 1$, we can approximate the transfer time as: $T_0 = \pi/2|b|$. To calculate $\mathcal{G}$ (Eq. 5 main text) to first order, we must first compute the next-order correction for the lowest energy eigenstates

$$|\psi^1_\pm\rangle = \sum_n \frac{\langle x_n|H'|\psi^0_\pm\rangle}{\mu - \epsilon_n\mathcal{J}_0\left(\frac{Ea_0}{\omega}\right)}|x_n\rangle \, . \tag{24}$$

We then have

$$|\psi_\pm\rangle = |\psi^0_\pm\rangle + \sum_n^{N-2} \frac{\mathcal{J}_0\left(\frac{Ea_0}{\omega}\right)}{\mu - \epsilon_n \mathcal{J}_0\left(\frac{Ea_0}{\omega}\right)} \sqrt{\frac{1}{N-1}} \left[\sin\left(\frac{n\pi}{N-1}\right) \pm \sin\left(\frac{n(N-2)\pi}{N-1}\right)\right] |x_n\rangle \tag{25}$$

which must be normalized, yielding

$$|\psi_\pm\rangle^{\rm n} = \frac{|\psi_\pm\rangle}{|\langle\psi_\pm|\psi_\pm\rangle|^{1/2}}; \qquad \mathcal{G}[|\psi_\pm\rangle^{\rm n}] = |\langle 1|\psi_\pm\rangle^{\rm n}\langle N|\psi_\pm\rangle^{\rm n}| = \frac{1}{2|\langle\psi_\pm|\psi_\pm\rangle|} \tag{26}$$

If we approximate $\frac{\mathcal{J}_0\left(\frac{Ea_0}{\omega}\right)}{\mu - \epsilon_n \mathcal{J}_0\left(\frac{Ea_0}{\omega}\right)} \simeq \frac{\mathcal{J}_0\left(\frac{Ea_0}{\omega}\right)}{\mu}$, noting that:

$$\sum_{n=1}^{N-2} \left\{\sin\left(\frac{n\pi}{N-1}\right) \pm \sin\left(\frac{n(N-2)\pi}{N-1}\right)\right\}^2 = N-1, \quad \forall N > 3 \tag{27}$$

finally we find

$$\mathcal{G}[|\psi_\pm\rangle^{\rm n}] = \frac{1}{2 + \frac{2}{\mu^2}\mathcal{J}_0^2\left(\frac{Ea_0}{\omega}\right)} \ . \tag{28}$$

Clearly in the limit of $\mathcal{J}_0\left(\frac{Ea_0}{\omega}\right) \to 0, \quad \mathcal{G}[|\psi_\pm\rangle^{\rm n}] \to 1/2$. This implies that the localization of the eigenstates at the edges increases, and therefore the long-range dynamics is particularly clean, although slower. As the Bessel function approaches a zero, the atoms become disconnected because the tunneling rates are renormalized to zero.

[1] P. Delplace, D. Ullmo and G. Montambaux, *Zak phase and the existence of edge states in graphene*, Phys. Rev. B. **84**,195452 (2011)

[2] Marcos Atala *et al.*, *Direct measurement of the Zak phase in topological Bloch bands*, Nature 10.1038 (2013)

[3] K. Winkler *et al*, *Repulsively bound atom pairs in an optical lattice*, Nature 10.1038 (2006)

[4] C. E. Creffield and G. Platero, *Localization of two interacting electrons in quantum dot arrays driven by an ac field*, Phys. Rev. B, **69**, 165312 (2004); *Coherent control of interacting particles using dynamical and Aharonov-Bohm Phases*, Phys. Rev. Lett. **105**,086804 (2010).

[5] M. Bukov, L. D'Alessio and A. Plokovnikov, *Universal high-frequency behavior of periodically driven systems: from dynamical stabilization to Floquet engineering*, Advances in Physics, 2015, Vol. 64, No. 2, 139-226

[6] A. Eckardt and E. Anisimovas, *High-frequency approximation for periodically driven quantum systems from a Floquet-space perspective*, New J. Phys **17** 093039 (2015)

[7] T. Mikami *et al.*, *Brillouin-Wigner theory for high-frequency expansion in periodically driven systems: Application to Floquet topological insulators*, arXiv:1511.00755
